# A pilot randomized controlled trial of an online mindfulness-based intervention to reduce patient anxiety before a first-time screening colonoscopy

**DOI:** 10.1007/s10552-026-02129-0

**Published:** 2026-03-03

**Authors:** Brent Emerson, Paul L. Reiter, Abigail B. Shoben, Maryanna Klatt, Subhankar Chakraborty, Mira L. Katz

**Affiliations:** 1 Division of Health Behavior and Health Promotion, College of Public Health, The Ohio State University, 1841 Neil Avenue, Columbus, OH 43210 USA; 2 Comprehensive Cancer Center, The Ohio State University, Columbus, OH USA; 3 Division of Biostatistics, College of Public Health, The Ohio State University, Columbus, OH USA; 4 Department of Family and Community Medicine, Center for Integrative Health, College of Medicine, The Ohio State University, Columbus, OH USA; 5 Department of Internal Medicine, College of Medicine, The Ohio State University, Columbus, OH USA

**Keywords:** Colorectal cancer, Screening, Mindfulness, Mindfulness-based intervention, Anxiety

## Abstract

**Purpose:**

Anxiety before a first-time screening colonoscopy is a commonly reported patient-level barrier to completing colorectal cancer (CRC) screening. This pilot study evaluated the feasibility, acceptability, and preliminary efficacy of “For peace of mind. Get Screened,” a brief online mindfulness-based intervention (MBI) to reduce anxiety before a first-time screening colonoscopy.

**Methods:**

Participants (n = 100) were randomized (October 2023—July 2024) to usual care or the MBI (daily CRC screening infographics guided by the Protection Motivation Theory, mindfulness infographics, and brief mindfulness meditations based on Monitor and Acceptance Theory) starting five days before their scheduled colonoscopy. Anxiety was assessed using the State-Trait Anxiety Inventory—State Subscale (STAI-S) in the endoscopy suite’s waiting room.

**Results:**

Participant retention was 83% due to patient and health system issues. Participant satisfaction with the MBI was high (mean: 6.40 out of 7). The MBI group had an average STAI-S score 2.54 points lower than the usual care group, though this difference was not statistically significant (32.24 vs. 34.78; p = 0.21). However, among participants not lost to follow up (n = 83), the MBI group had significantly lower waiting room STAI-S scores compared to the usual care group (31.52 vs. 35.21; p = 0.02). Clinical outcomes did not differ between groups.

**Conclusion:**

Feasibility of the MBI was partially limited due to logistical issues. Findings support MBI acceptability and suggest that a brief MBI has the potential to decrease anxiety prior to a first-time colonoscopy. A larger randomized controlled trial is needed to further examine the efficacy of this intervention.

**Clinical trial registration:**

ClinicalTrials.gov NCT06233253.

## Introduction

Colorectal cancer (CRC) is the third most common cancer (excluding skin cancer), and second leading cause of cancer-related deaths among males and females in the United States (U.S.) [1]. CRC screening is cost-effective in reducing CRC incidence and mortality rates [2–4], and a colonoscopy is considered the gold standard of CRC screening because of its ability to prevent or diagnose cancer [5]. The United States Preventive Services Task Force (USPSTF) recommends CRC screening for asymptomatic adults starting at age 45 who are at average risk for CRC (i.e., no prior diagnosis of CRC, adenomatous polyps, or inflammatory bowel disease; no personal diagnosis or family history of Lynch syndrome or familial adenomatous polyposis) [6]. Despite CRC screening being recommended and available in the U.S., the percentage of age-eligible adults up-to-date with CRC screening was only 59% in 2021 [7].

Pre-procedural anxiety is a common patient-level barrier to completing a colonoscopy with studies reporting more than 50% of patients having moderate-to-severe anxiety [8–10]. Anxiety prior to a colonoscopy is attributed to the difficult preparation process, concern about the invasiveness of the procedure, embarrassment, pain/discomfort or complications from the procedure, and a potential cancer diagnosis [8]. Pre-procedural anxiety may impact clinical and patient outcomes, including cancelation or no-show rates [11, 12], bowel preparation tolerance and quality [13], the amount of sedatives needed during the procedure [14], procedural time [15], and patient satisfaction [16].

To reduce anxiety before a colonoscopy, previous research has explored using educational materials [17], patient navigation [18], music [19], breathing exercises [20], guided imagery [20], aromatherapy [21], hypnosis [22], and white noise [23]. However, little is known about the use of mindfulness before a colonoscopy. Mindfulness is a form of meditation that focuses on staying within the present moment to reduce anxiety [24]. Previous mindfulness research about cancer-related topics has focused primarily on individuals receiving cancer treatment or during survivorship as a way to decrease perceived stress, fear of cancer recurrence, and improve quality of life [25]. However, there is a lack of randomized controlled trials (RCTs) evaluating mindfulness-based interventions (MBIs) to reduce anxiety prior to cancer screening [26].

Given that anxiety is commonly reported by patients prior to undergoing a colonoscopy, we developed the “For peace of mind. Get Screened,” MBI. As previously described [27], this is a brief online MBI that was designed to reduce pre-procedural anxiety among average-risk adults before a first-time screening colonoscopy. In the current manuscript, we report the results of a pilot study that examined the feasibility, acceptability, and preliminary efficacy of this MBI. We hypothesized the following: 1) we would recruit and randomize 100 eligible adults and achieve a retention rate of 90% (i.e., intervention feasibility); 2) participants randomized to the MBI would report an average satisfaction score with the intervention ≥ 6.0, measured using a 7-point Likert scale (i.e., intervention acceptability); and 3) participants randomized to the MBI would report less anxiety than those in the usual care group (i.e., preliminary efficacy data).

## Methods

### Participants and procedures

Patient recruitment occurred between October 2023 and July 2024 in collaboration with practicing gastroenterologists. Study staff reviewed medical charts of patients scheduled for a colonoscopy to identify and contact potentially eligible patients. Patients were eligible if they: 1) were ages 45–75; 2) were at average-risk for CRC [6]; 3) had scheduled their first-time screening colonoscopy as an outpatient at least 14 days in advance; 4) were able to speak and read English; 5) had daily access to a working telephone, email address, and internet connection; and 6) provided informed consent. Exclusion criteria included having: 1) had a previous colonoscopy; 2) a scheduled colonoscopy for diagnostic purposes; 3) auditory or visual impairment that prevented internet use; 4) a previous cancer diagnosis (other than skin cancer); 5) a previous diagnosis of a mental health disorder; 6) mental health treatment during the time of the study; 7) a family history of CRC; 8) a personal history of inflammatory bowel disease (ulcerative colitis or Crohn’s disease); or 9) a confirmed or suspected hereditary CRC syndrome, such as familial adenomatous polyposis or Lynch syndrome. The study was approved by The Ohio State University’s Institutional Review Board, and registered on clinicaltrials.gov (NCT06233253).

Once contacted, study staff verified eligibility and obtained informed consent and HIPAA research authorization. After completing the consent process, individuals completed a baseline survey using a link sent in an email, and then they were randomized to the MBI or usual care using a 1:1 randomization scheme. To effectively administer group-specific activities (i.e., mindfulness meditations), study staff and participants were not blinded to group allocation.

### Study groups

Study group-specific activities began five days before the participant’s scheduled colonoscopy to align with the start of dietary changes before a screening colonoscopy. A start date reminder was sent to participants via email/text three days and one day before the start of study activities that were accessed from a group-specific landing page. Each day, if the participant did not complete the study group-specific activity by 5:00 pm, one email/text reminder was automatically sent to the participant.

## MBI

On each of the five days leading up to the day of the scheduled colonoscopy, participants in the MBI group were provided access to a: 1) CRC screening infographic (guided by the Protection Motivation Theory); 2) mindfulness infographic; and 3) brief mindfulness meditation (5 min or less and scripts based on Monitor and Acceptance Theory). After each mindfulness meditation, participants completed a brief post-meditation assessment which included measures of state mindfulness (i.e., perceived distraction during the meditation) and perceived satisfaction with the meditation.

On Day 1, participants selected their preferred meditation voice (white male or female, black male or female) and viewed the “Meet the Study Team” infographic which included a photo of the study staff who would greet them in the endoscopy waiting room. On Days 2–4, participants had access to a CRC screening and mindfulness infographic, a mindfulness meditation, and completed a post-meditation assessment. On Day 5, participants received a reminder to complete study activities (i.e., CRC screening and mindfulness infographics, bowel preparation mindfulness meditation, post-meditation assessment) halfway through bowel preparation, and participants were instructed to complete the bowel preparation survey before and after the mindfulness meditation. The day of the colonoscopy, study staff greeted the participant in the endoscopy waiting room, and provided the participant with an encrypted iPad, and disposable headphones to complete the brief mindfulness meditation (≈ 5 min), post-meditation assessment, and a short waiting room survey. The day after their colonoscopy, participants were emailed a link to a follow-up survey. If a participant did not complete the follow-up survey, a maximum of three reminders were sent via email/text with one reminder every two days.

## Usual care

On Day 1, participants in the usual care group were sent an email with a link to the usual care landing page which provided participants access to the “Meet the Study Team” infographic, and a reminder that their next study activity will occur in four days during bowel preparation. On Day 5, participants received a reminder to complete the bowel preparation survey halfway through bowel preparation. The day of the colonoscopy, study staff greeted the participant in the endoscopy waiting room, and after a brief 5-min waiting period, provided the participant with an encrypted iPad to complete the endoscopy waiting room survey. The day after their colonoscopy, participants were emailed a link to a follow-up survey. If a participant did not complete the follow-up survey, a maximum of three reminders were sent via email/text with one reminder every two days. After completing the follow-up survey, participants in the usual care group were granted access to the CRC screening and mindfulness infographics and select mindfulness meditations via email.

### Lost to follow up

Participants were considered lost to follow up if they canceled or rescheduled their colonoscopy during the study period or if they were inactive, which was defined as missing two consecutive days of study activities. If a participant was considered lost to follow up, they were sent an email with a link to a survey to assess the primary reason for loss to follow up (e.g., cancelling procedure), and they did not receive access to subsequent study activities. If a participant did not complete the loss to follow-up survey, a maximum of three reminders were sent via email/text with one reminder every two days. Participants were emailed an electronic gift card after completing the lost to follow-up survey.

### Measures

Data were collected using online surveys at baseline, during bowel preparation, in the waiting room, follow up or at lost to follow up, and a post-colonoscopy medical chart review. Participants in both groups received electronic gift cards for completing each online survey (maximum of $90 for completing all surveys).

## Participant surveys

### Participant characteristics

Participant characteristics were assessed during the baseline survey and included age, gender, race and ethnicity, educational attainment, annual household income, and health insurance status. Health literacy was assessed using the Single Item Health Literacy Scale (SILS) to assess the frequency an individual requires additional help to read written material from their doctor or pharmacy. Response options were “never,” “rarely,” “sometimes,” “often,” and “always” [28]. We categorized participants as having adequate (never or rarely) or limited (sometimes, often, always) health literacy [28]. Measures of clinical and behavioral history were also assessed during the baseline survey including narcotic use, anti-anxiety medication use, and previous meditation experience.

### Pre-procedural anxiety

Pre-procedural anxiety was measured using the State-Trait Anxiety Inventory-State Subscale (STAI-S). The STAI-S uses a 4-point Likert scale to assess intensity of current feelings (not at all, somewhat, moderately so, very much so) [29]. Scores range from 20–80 with higher scores indicating higher levels of state anxiety [29].

### Attitudes

Attitudes about CRC and CRC screening were measured on a 5-point Likert scale (strongly disagree to strongly agree), and were guided by the Protection Motivation Theory including: 1) perceived severity of CRC; 2) perceived susceptibility of CRC; 3) self-efficacy to: complete the bowel preparation, complete the colonoscopy, and prevent CRC; 4) response efficacy; 5) response costs; 6) intrinsic and extrinsic rewards; and 7) fear/worry of CRC and of the colonoscopy [30].

### Mindfulness

The Philadelphia Mindfulness Scale (PHLMS) is a validated self-report instrument designed to assess two core components of mindfulness: awareness and acceptance. The PHLMS consists of 20-items, divided equally into two subscales: 10 items for awareness (measuring present-moment attention to internal and external experiences) and 10 items for acceptance (measuring nonjudgmental openness to experience) [31]. The subscales of the PHLMS are commonly used to assess Monitor and Acceptance Theory constructs of monitoring (i.e., awareness) and acceptance [31, 32]. Total PHLMS scores range from 20–100 with higher scores indicating increased trait mindfulness [31]. Similarly, subscale scores range from 10–50 with higher scores indicating increased awareness and acceptance [31].

### Post-meditation assessment (MBI group only)

After each mindfulness meditation, participants in the MBI group were asked to rate their state mindfulness (perceived distraction during meditation) using a visual analog scale (VAS) (0 = Not distracted – 10 = Very distracted), and perceived satisfaction with the meditation using a 5-point Likert scale (very dissatisfied, somewhat dissatisfied, neither dissatisfied nor satisfied, somewhat satisfied, very satisfied).

### Feedback about research participation and colonoscopy experience

Participants were asked to provide any thoughts, comments, or feedback about their participation in the research study and/or about their colonoscopy experience as an open-ended response in the follow-up survey.

### Post-colonoscopy medical chart review

A measure of bowel preparation quality was documented using the Boston Bowel Preparation Scale (BBPS) or a 5-point Likert scale (unsatisfactory, inadequate, fair, good, excellent), and BBPS scores range from 0 (unprepared colon) to 9 (perfectly clean colon) [33]. Additional measures collected from the participants’ medical charts included biometrics (heart rate, blood pressure, height, and weight) at time of check-in to the endoscopy holding suite, sedation metrics (medication and dose delivered), procedural metrics (cecal intubation, procedural time, total time in endoscopy suite, and perceived difficulty), and findings (number, size, and location of polyps and diagnosis).

### Feasibility and acceptability

Feasibility was assessed using the following measures: 1) the number of potentially eligible patients identified via medical chart review; 2) the number of potentially eligible patients contacted; 3) the number of participants recruited; 4) the number of participants lost to follow up; 5) the amount of time (months) necessary to reach the recruitment goal; and 6) the amount of missing anxiety data (i.e., STAI-S). Participant’s acceptability of the MBI was assessed using the following measures: 1) study access (engagement with emailed link to daily activity); 2) perceived benefit of the mindfulness meditations (yes/no); and 3) perceived satisfaction with the MBI (1 = Very dissatisfied – 7 = Very satisfied).

### Sample size

A sample size calculation was conducted to estimate the number of participants necessary to detect a difference in pre-procedural anxiety, measured using the STAI-S in the endoscopy waiting room between study groups. We used a two-sided alpha = 0.05, power = 0.8, and an estimated average (standard deviation: SD) decrease in STAI-S scores = 5.1 (0.53). The calculation determined that 90 participants were needed to adequately power the pilot RCT, and we enrolled 100 participants to account for loss to follow up.

### Data analysis

#### Quantitative data

We calculated descriptive statistics for all variables, and statistical tests used a p < 0.05 to determine statistical significance. For scales requiring complete item responses (e.g., STAI, PHLMS), missing items were imputed using within-person mean imputation.

A modified intention to treat (mITT) analysis was conducted to assess differences in endoscopy waiting room STAI-S scores between study groups, and all available STAI-S data were included in a generalized linear mixed model (GLMM) to preserve the randomized sample [34, 35]. The GLMM included STAI-S scores collected at baseline, during bowel preparation (post mindfulness meditation in the MBI group and usual care), and in the endoscopy waiting room. Fixed effects included time point indicators, study group, and interaction terms between study group and baseline and bowel preparation time points. A random effect for participant ID was included to account for within-subject correlations, and an identity link function was used assuming a linear relationship between predictors and outcome. A per-protocol secondary analysis was conducted to assess differences in STAI-S scores between study groups among active participants.

A chi-square test of independence was used to assess statistically significant difference between categorical variables. An independent samples t-test were used to compare continuous outcomes between study groups, and a paired measures t-test was used to compare changes in the continuous outcomes among the same participants at baseline and follow up. Analyses were performed with STATA/IC 15 statistical software.

### Qualitative data

Responses to open-ended survey items were entered into and analyzed using NVivo software. Primary codes were created and inductive coding was used to create subcodes under each primary code that corresponded to participant responses. Transcripts were analyzed more than once to refine subcodes and ensure data saturation. The results were reviewed by study team members to ensure accurate coding and interpretation.

## Results

**Fig. 1:**
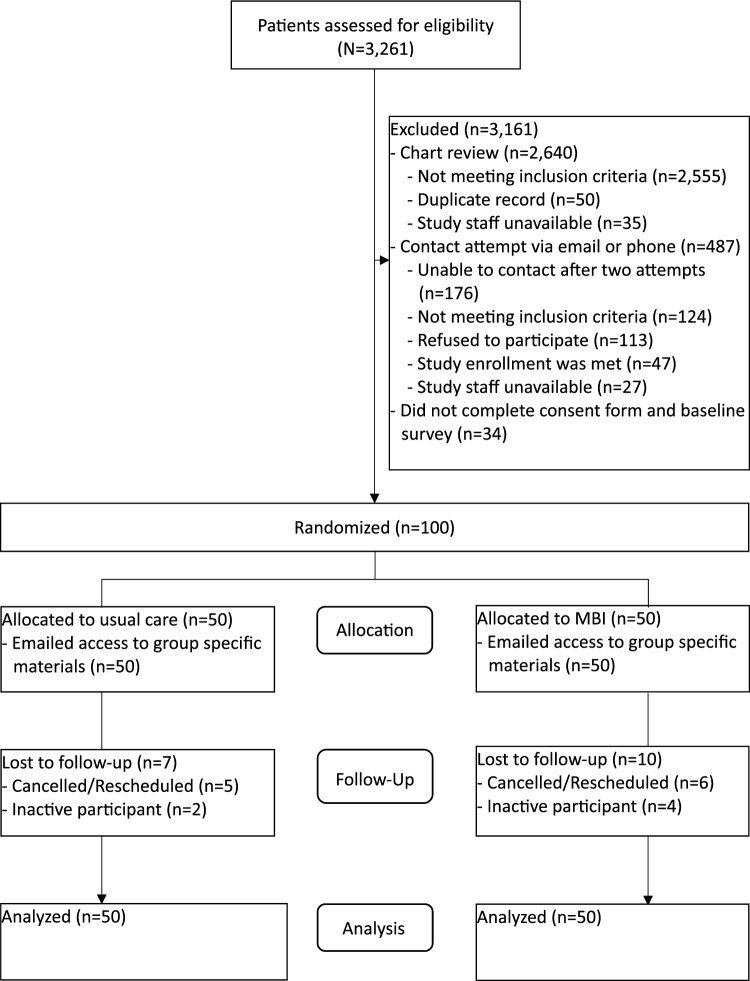
CONSORT Diagram

The CONSORT diagram is provided in Fig. 1, and 100 participants were randomized between October 2023 and July 2024. We screened 3,261 patient records for eligibility, and 2,640 patients were excluded during medical chart review. Of the 621 patients eligible for contact, 487 patients were excluded including 113 patients who refused to participate mainly due to lack of interest in the research or being too busy. An informed consent and HIPAA research authorization form was sent to the 134 interested and eligible patients, and 105 (78%) participants signed and returned the form. Of the 105 patients who were sent the baseline survey, 100 completed the survey in the appropriate timeframe and were randomized to usual care or the MBI. Of the 100 randomized participants, 17 participants were lost to follow up due to either a canceled/ rescheduled colonoscopy (n = 11) or study inactivity (n = 6); however, baseline STAI-S scores from all participants were included in the analysis.

### Participant characteristics

The baseline characteristics of the 100 randomized participants by study group is provided in Table 1. The average (SD) age of participants was 49.8 (5.4) years. The majority of participants were female (52%), white (73%), and non-Hispanic (89%). Many participants had a four-year college degree or graduate/professional degree (66%), health insurance (96%), a household income greater than $100,000 (64%), and adequate health literacy (93%). A few participants reported use of narcotics (2%) or anti-anxiety medication (8%), and about half of participants reported previous meditation experience (47%).

**Table 1:** Baseline Participant Characteristics by Study Group (n = 100)

	Usual care (n = 50)	MBI (n = 50)
	n (%)	
Age: average years (SD)	49.8 (5.7)	49.9 (5.1)
*Gender*		
Male	21 (42)	24 (48)
Female	27 (54)	25 (50)
Missing	2 (4)	1 (2)
*Race*		
White	40 (80)	33 (66)
Black or African American	5 (10)	8 (16)
Asian	4 (8)	2 (4)
American Indian or Alaska Native	0 (0)	1 (2)
More than one	0 (0)	3 (6)
Other	1 (2)	3 (6)
*Ethnicity*		
Hispanic	3 (6)	2 (4)
Non-Hispanic	44 (88)	45 (90)
Missing	3 (6)	3 (6)
*Educational attainment*		
Less than high school	0 (0)	0 (0)
High school/GED	7 (14)	11 (22)
Two-year college or technical degree	8 (16)	7 (14)
Four-year college degree	11 (22)	14 (28)
Graduate/Professional degree	24 (48)	17 (34)
Missing	0 (0)	1 (2)
*Household income*		
Less than $20,001	0 (0)	3 (6)
$20,001-$40,000	0 (0)	6 (12)
$40,001-$60,000	5 (10)	2 (4)
$60,001-$80,000	2 (4)	5 (10)
$80,001-$100,000	7 (14)	6 (12)
Greater than $100,000	36 (72)	28 (56)
*Health insurance*		
Yes	49 (98)	47 (94)
No	0 (0)	1 (2)
I don’t know	0 (0)	1 (2)
Missing	1 (2)	1 (2)
Health literacy		
Adequate	47 (94)	46 (92)
Limited	2 (4)	3 (6)
Missing	1 (2)	1 (2)
*Use of narcotics*		
Yes	0 (0)	2 (4)
No	50 (100)	46 (92)
I don’t know	0 (0)	2 (4)
*Use of anti-anxiety medication*		
Yes	3 (6)	5 (10)
No	47 (94)	44 (88)
I don’t know	0 (0)	1 (2)
*Meditation experience*		
Yes	22 (44)	25 (50)
No	28 (56)	19 (38)
I don’t know	0 (0)	6 (12)

*SD* Standard deviation, *MBI* Mindfulness-based intervention, *GED* General education development

### Pre-procedural anxiety

**Fig. 2:**
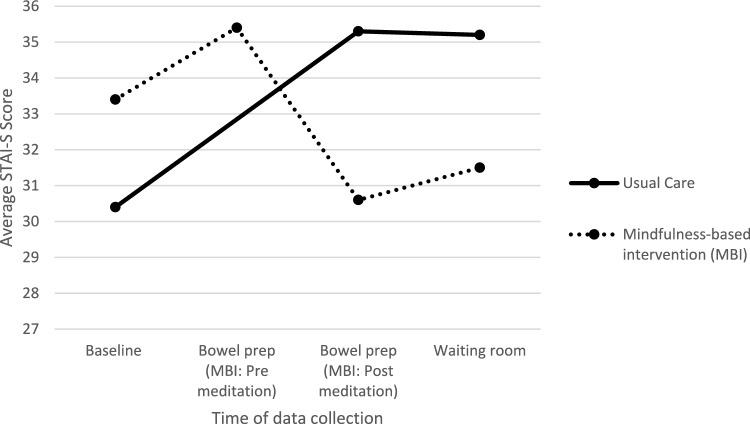
Average State-Trait Anxiety Inventory-State Subscale (STAI-S) Scores at Baseline, during Bowel Prep, and in Endoscopy Waiting Room by Study Group (n = 100)

A depiction of mean STAI-S scores including data from all participants is included in Fig. 2. As estimated by the GLMM, those in the MBI group had a higher average baseline STAI-S score (33.36, SD = 11.18) when compared to those in the usual care group (30.42, SD = 7.92); however, baseline differences may be attributed to random chance due to randomization. Those randomized to the MBI group had an average endoscopy waiting room STAI-S score that was 2.54 (95% CI: -6.49, 1.42) points lower than those randomized to the usual care group and this difference was not statistically significant (p = 0.21; 95% CI: 6.49 points lower to 1.42 points higher). When comparing within group average baseline STAI-S scores and endoscopy waiting room STAI-S scores, those in the MBI group had an average endoscopy waiting room STAI-S score that was − 1.28 (95% CI: − 3.96, 1.40) points lower than baseline (not statistically significant; p = 0.35); however, those in the usual care group had an average endoscopy waiting room STAI-S score that was 4.53 (95% CI: 1.95, 7.11) points higher than baseline (statistically significant; p < 0.01). Thus, the interaction comparing the difference between baseline and endoscopy waiting room STAI-S scores by study group was 5.81 (95% CI: 2.08, 9.54) indicating a statistically significant group by time treatment effect (p < 0.01). Similarly, among participants not lost to follow up (n = 83), the MBI group had significantly lower waiting room STAI-S scores when compared to the usual care group (31.52 vs. 35.21, respectively; p = 0.02) when using an independent samples t-test.

### Attitudes

There were no statistically significant changes in perceived susceptibility, perceived severity, self-efficacy for completing bowel preparation or for completing a colonoscopy, response efficacy, or response costs when comparing baseline and follow up scores in either group (p > 0.05). There was a statistically significant decrease in average fear/worry of colonoscopy scores when comparing baseline and follow up among those in the MBI group (− 0.60; p < 0.01) and the usual care group (− 0.65; p < 0.01). However, only those in the MBI group had a significant increase in self-efficacy for CRC prevention (0.68; p < 0.01), and a significant decrease in intrinsic rewards (− 0.43; p < 0.01) and extrinsic rewards (-0.30; p = 0.01) when comparing baseline and follow up.

### Mindfulness

There were no statistically significant differences in average total PHLMS scores at follow up when comparing active participants in the MBI and usual care group (73.71 vs. 75.17; p = 0.25). There were statistically significant increases when comparing average baseline and follow up awareness scores for active participants in the MBI group (37.84 vs. 39.15; p = 0.02) and usual care group (37.64 vs. 39.55; p = 0.01); however, there were no statistically significant differences when comparing baseline and follow up acceptance scores for those in the MBI group or usual care group (p > 0.05).

### Loss to follow up

A total of 17 participants were lost to follow up from the MBI group (n = 10) and usual care group (n = 7). Of the 11 cancelled/rescheduled colonoscopies, six participants’ procedures were cancelled by medical staff because of medical staff illness, patient illness, and health insurance issues. Four participants cancelled their colonoscopies due to personal emergencies, concern about finding cancer, and insurance issues. One colonoscopy was cancelled without documentation of who or why the procedure was cancelled. Six participants lost to follow up were due to participant inactivity because of technology issues and personal emergencies. The other three participants did not complete the survey and were inactive for unknown reasons. Those lost to follow up were not statistically different (p > 0.05) than active participants when comparing mean age, gender, ethnicity, education, household income, or health literacy; however, there was a statistically significant difference (x^2^ = 4.18, p = 0.04) when comparing race (53% vs. 77% white, respectively) indicating a higher percentage of non-white participants who were lost to follow up when compared to active participants.

### Feedback about research participation and colonoscopy experience

Participants reported that the study was well organized, and that communication was clear and concise. Participants reported a perceived benefit from the mindfulness meditations, and recommended ways to improve the research study by increasing the mindfulness meditation length, allowing multiple views of each mindfulness meditation, and providing the ability to customize the voice, background image and audio of the mindfulness meditation.

Overall, participants reported a positive colonoscopy experience, particularly when engaging with the medical staff. A few participants reported a negative experience due to ineffective bowel preparation and an adverse event during the colonoscopy. Participants suggested ways to improve the colonoscopy experience by providing better communication and education to patients about the bowel preparation and the colonoscopy.

### Medical chart review

There was no statistically significant difference when comparing bowel preparation quality (BBPS or Likert scale), biometrics (heart rate, blood pressure) at time of check-in to the endoscopy holding suite, sedation metrics (medication and dose delivered), procedural metrics (cecal intubation, procedural time, total time in endoscopy suite, and perceived difficulty), and findings (number, size, and location of polyps, and diagnosis) between study groups (p > 0.05).

### Feasibility and acceptability

Study staff assessed 3,261 medical charts for eligibility, and 487 patients were eligible for contact. The study team was able to recruit 100 participants in a ten month period (October 2023—July 2024) with 17 (17%) participants lost to follow up. Endoscopy waiting room STAI-S scores was collected in all 83 active participants with only seven missing items replaced using data imputation.

The majority (> 81%) of active participants accessed the assigned study activities each day of the study. Most active participants in the MBI group believed that the mindfulness mediations were helpful during bowel preparation (34/40; 85%) and before their colonoscopy (38/40; 95%). Average satisfaction with the MBI was high (6.40 of 7), and 98% would recommend the MBI to others.

## Discussion

Anxiety is commonly reported by patients prior to undergoing a colonoscopy, and little is known about the use of mindfulness to reduce anxiety before a colonoscopy. Thus, the “For peace of mind. Get Screened,” MBI was developed to reduce pre-procedural anxiety among average-risk adults before a first-time screening colonoscopy [27]. This pilot study examined the feasibility, acceptability, and preliminary efficacy of the MBI, and yielded five key findings.

First, the implementation of the MBI was partially feasible. We recruited 100 participants in a ten month period (October 2023—July 2024) with only 17 (17%) participants lost to follow up (hypothesis partially supported). Endoscopy waiting room STAI-S scores was collected in all 83 active participants with only seven missing items replaced using data imputation. Similar to other clinical trials, racial and ethnic minorities were more likely to be loss to follow up [36, 37]; however, this is difficult to interpret due to the frequency of colonoscopies being cancelled by the healthcare system. Future research evaluating MBIs should prioritize the recruitment of participants from diverse racial and ethnic backgrounds. Furthermore, this study considered participants lost to follow up if they did not complete two consecutive days of study related activities. This decision was made to explore the effectiveness of the MBI when comparing active participants with consistent MBI access. Future RCTs to reduce pre-procedural anxiety before a first-time screening colonoscopy should not include study inactivity as a criterion for loss to follow up, and report inactivity as part of the process evaluation.

Second, participants reported high levels of satisfaction with the MBI (hypothesis supported), and most participants would recommend the MBI to a family member or friend. Participants also provided the following recommendations to improve the mindfulness meditations before testing the intervention in a large RCT: 1) increase the length of the mindfulness meditations; 2) increase the number of voices used to record the mindfulness meditations; 3) allow participants to select their preferred background image and sound; 4) allow the mindfulness meditations to be watched multiple times; and 5) move the endoscopy waiting room meditation to a quiet space with less distractions.

Third, there was a significant difference in endoscopy waiting room STAI-S scores when comparing active participants in the MBI and the usual care group. The lack of difference in endoscopy waiting room STAI-S scores between groups when including all participants suggest the MBI was not effective (hypothesis not supported). However, when assessing the interaction between baseline and endoscopy waiting room STAI-S scores and study groups, those in the usual care group had a statistically significant increase in average STAI-S scores while those in the MBI group had a non-statistically significant decrease in STAI-S scores. Thus, the MBI may help prevent an increase in pre-procedural anxiety during critical moments leading up to a first-time screening colonoscopy [8]. These preliminary findings suggest a positive effect, and provide important data to inform the planning of a larger RCT, including sample size calculations and power estimates, comparing mindfulness to alternative stress-reduction interventions (e.g., music, breathing exercises).

Fourth, the MBI did not significantly improve colonoscopy-related outcomes. Among participants who received a BBPS score, the average bowel preparation quality was 8.82 out of 9, indicating excellent bowel preparation, and exceeding the expected average of 6.0 [33]. Selection bias may explain the lack of variation in bowel preparation scores, as those who are willing to participate in the study may be different than those who refused participation. Additionally, participants were informed of the purpose of the study during the informed consent process; therefore, the results may be subject to demand characteristic biases as participants may have changed their behavior to align with study expectations. As expected with satisfactory BBPS scores, there were no differences in procedure time, cecal intubation time, and procedural difficulty when comparing those in the MBI group and usual care group. Additionally, there was no significant difference when comparing heart rate and blood pressure at the time of admission to the endoscopy holding suite, and sedation dose delivered before the participant’s first-time screening colonoscopy between those in the MBI group and usual care group. A limitation of this study included implementing the mindfulness meditation in the endoscopy waiting room instead of in the endoscopy suite’s holding area to mitigate impact on workflow. Future research to explore the effectiveness of the MBI on heart rate and blood pressure at the time of admission to the endoscopy suite, and sedation dose delivered should consider providing the mindfulness meditations to the patients in the holding area in the endoscopy suite, if possible.

Fifth, despite satisfactory BBPS scores, participants reported that there was a need to improve patient education and communication regarding bowel preparation. One participant in the MBI group reported not knowing they had to change their diet five days before their colonoscopy until they reviewed the educational infographic (“Honestly, I got more out of this study than I did out of any communication or preparation materials from the doctor’s office. Without this study telling me on day 1 (five days before the colonoscopy) that I should check the instructions from my doctor, I would never have known I needed to start so early. The doctor’s office never sent any information. I had to search for all the prep instructions on MyChart (a huge miss, if you ask me.)”). Future research should aim to improve communication about the necessary dietary changes starting five days before the colonoscopy.

This pilot study has several strengths. The study team was able to recruit and randomize 100 participants, and all active participants were able to complete the endoscopy waiting room STAI-S before their first-time screening colonoscopy. Participants in the MBI group reported high levels of satisfaction with the MBI, and almost all participants would recommend the MBI to a family member or friend before their colonoscopy. This study was able to demonstrate intervention safety, and no adverse events were reported during the study. Lastly, areas of improvement for the MBI were identified by participants prior to testing the intervention in a large RCT.

However, this study had several limitations. A limitation of online interventions is the inability to ensure participants are adhering to online intervention activities as intended. Although some measures of study engagement can be measured (e.g., number of study activities accessed, number surveys completed), it is possible participants were not completing activities as intended. There was also potential for response bias while completing study surveys. To encourage participants to respond honestly and reduce response bias, survey instructions reminded participants that there was no right or wrong answer, and all data would be de-identified before analysis. In addition, there was potential contamination between study groups as two participants, one in the MBI group and one in usual care, resided in the same household, and future studies should consider limiting eligibility to one participant per household. This study was limited to one large health system located in the Midwest which may limit generalizability of findings to other populations. Lastly, a limitation of the study is the use of mITT analysis and future studies should not exclude participants based on inactivity.

Findings support the feasibility and acceptability of the MBI and suggest that a brief MBI may help reduce anxiety prior to a first-time colonoscopy. Future research should evaluate the effectiveness of the “For peace of mind. Get Screened” MBI in a larger and more diverse sample, and compare it to an alternative stress-reduction intervention (e.g., music, breathing exercise) to better understand its relative effectiveness.

## Data Availability

The datasets generated and/or analyzed during the current study are available from the corresponding author upon reasonable request.

## Code availability

The STATA/IC 15 code used during the current study is available from the corresponding author upon reasonable request.

## Declarations

This manuscript is part of Brent Emerson’s (first author) dissertation research.

## References

[1] Siegel RL, Kratzer TB, Giaquinto AN, Sung H, Jemal A (2025) Cancer statistics, 2025. CA Cancer J Clin 75:10–45. 10.3322/caac.21871 39817679 10.3322/caac.21871 PMC11745215

[2] Guy GP Jr., Richardson LC, Pignone MP, Plescia M (2014) Costs and benefits of an organized fecal immunochemical test-based colorectal cancer screening program in the United States. Cancer 120:2308–2315. 10.1002/cncr.28724 24737634 10.1002/cncr.28724 PMC4593052

[3] Lansdorp-Vogelaar I, Knudsen AB, Brenner H (2011) Cost-effectiveness of colorectal cancer screening. Epidemiol Rev 33:88–100. 10.1093/epirev/mxr004 21633092 10.1093/epirev/mxr004 PMC3132805

[4] Meester RGS, Doubeni CA, Zauber AG et al (2015) Public health impact of achieving 80% colorectal cancer screening rates in the United States by 2018. Cancer 121:2281–2285. 10.1002/cncr.29336 25763558 10.1002/cncr.29336 PMC4567966

[5] Nierengarten MB (2023) Colonoscopy remains the gold standard for screening despite recent tarnish. Cancer 129:330–331. 10.1002/cncr.34622 36602936 10.1002/cncr.34622

[6] Davidson KW, Barry MJ, Mangione CM et al (2021) Screening for colorectal cancer: US preventive services task force recommendation statement. JAMA 325:1965–1977. 10.1001/jama.2021.6238 34003218 10.1001/jama.2021.6238

[7] American Cancer Society (2023) Colorectal cancer facts & figures 2023–2025.

[8] Yang C, Sriranjan V, Abou-Setta AM, Poluha W, Walker JR, Singh H (2018) Anxiety associated with colonoscopy and flexible sigmoidoscopy: a systematic review. Am J Gastroenterol 113:1810–1818. 10.1038/s41395-018-0398-8 30385831 10.1038/s41395-018-0398-8 PMC6768596

[9] Niv Y, Bogolavski I, Ilani S et al (2012) Impact of colonoscopy on quality of life. Eur J Gastroenterol Hepatol 24:781–786. 10.1097/MEG.0b013e328352deff 22441512 10.1097/MEG.0b013e328352deff

[10] McEntire J, Sahota J, Hydes T, Trebble TM (2013) An evaluation of patient attitudes to colonoscopy and the importance of endoscopist interaction and the endoscopy environment to satisfaction and value. Scand J Gastroenterol 48:366–373. 10.3109/00365521.2012.758768 23320489 10.3109/00365521.2012.758768

[11] Bhise V, Modi V, Kalavar A et al (2016) Patient-reported attributions for missed colonoscopy appointments in two large healthcare systems. Dig Dis Sci 61:1853–1861. 10.1007/s10620-016-4096-3 26971093 10.1007/s10620-016-4096-3

[12] Lacy NL, Paulman A, Reuter MD, Lovejoy B (2004) Why we don’t come: patient perceptions on no-shows. Ann Fam Med 2:541–545. 10.1370/afm.123 15576538 10.1370/afm.123 PMC1466756

[13] Bessissow T, Van Keerberghen C-A, Van Oudenhove L et al (2013) Anxiety is associated with impaired tolerance of colonoscopy preparation in inflammatory bowel disease and controls. J Crohns Colitis 7:e580–e587. 10.1016/j.crohns.2013.04.011 23664621 10.1016/j.crohns.2013.04.011

[14] Sargın M, Uluer M (2020) The effect of pre-procedure anxiety on sedative requirements for sedation during upper gastrointestinal endoscopy. Turk J Surg 36:368–373. 10.47717/turkjsurg.2020.4532 33778396 10.47717/turkjsurg.2020.4532 PMC7963298

[15] Tam WW, Wong EL, Twinn SF (2008) Effect of music on procedure time and sedation during colonoscopy: a meta-analysis. World J Gastroenterol 14:5336–5343. 10.3748/wjg.14.5336 18785289 10.3748/wjg.14.5336 PMC2744067

[16] Bytzer P, Lindeberg B (2007) Impact of an information video before colonoscopy on patient satisfaction and anxiety - a randomized trial. Endoscopy 39:710–714. 10.1055/s-2007-966718 17661246 10.1055/s-2007-966718

[17] Hsueh F-C, Chen C-M, Sun C-A, Chou Y-C, Hsiao S-M, Yang T (2016) A study on the effects of a health education intervention on anxiety and pain during colonoscopy procedures. J Nurs Res. 10.1097/jnr.0000000000000112 26551213 10.1097/jnr.0000000000000112

[18] Rawl SM, Perkins SM, Tong Y et al (2024) Patient navigation plus tailored digital video disc increases colorectal cancer screening among low-income and minority patients who did not attend a scheduled screening colonoscopy: a randomized trial. Ann Behav Med 58:314–327. 10.1093/abm/kaae013 38470961 10.1093/abm/kaae013 PMC11008590

[19] Rudin D, Kiss A, Wetz RV, Sottile VM (2007) Music in the endoscopy suite: a meta-analysis of randomized controlled studies. Endoscopy 39:507–510. 10.1055/s-2007-966362 17554644 10.1055/s-2007-966362

[20] Albashir S, Durepos P, Causada Calo N et al (2021) Psychological interventions for reducing anxiety in patients undergoing first-time colonoscopy: a pilot and feasibility study. Eur J Gastroenterol Hepatol 33:e634–e641. 10.1097/meg.0000000000002186 34034274 10.1097/MEG.0000000000002186

[21] Hu PH, Peng YC, Lin YT, Chang CS, Ou MC (2010) Aromatherapy for reducing colonoscopy related procedural anxiety and physiological parameters: a randomized controlled study. Hepatogastroenterology 57:1082–1086 21410035

[22] Elkins G, White J, Patel P, Marcus J, Perfect MM, Montgomery GH (2006) Hypnosis to manage anxiety and pain associated with colonoscopy for colorectal cancer screening: case studies and possible benefits. Int J Clin Exp Hypn 54:416–431. 10.1080/00207140600856780 16950684 10.1080/00207140600856780

[23] Lazar Barzegar S, Mohammadi S, Shamsalinia A, Saberifar M (2023) The effects of white noise on preprocedural anxiety and vital signs among older adults undergoing colonoscopy. J Perianesth Nurs. 10.1016/j.jopan.2023.08.017 37966399 10.1016/j.jopan.2023.08.017

[24] Kabat-Zinn J (2003) Mindfulness-based stress reduction (MBSR). Constr Hum Sci 8:73–107

[25] Ledesma D, Kumano H (2009) Mindfulness-based stress reduction and cancer: a meta-analysis. Psycho-Oncol 18:571–579. 10.1002/pon.1400 10.1002/pon.1400 19023879

[26] Emerson B, Reddy M, Reiter PL et al (2024) Mindfulness-based interventions across the cancer continuum in the United States: a scoping review. Am J Health Promot 38:560–575. 10.1177/08901171241227316 38205783 10.1177/08901171241227316

[27] Emerson B, Reiter PL, Klatt M et al (2025) Development of a brief online mindfulness-based intervention to reduce patient anxiety before a first-time screening colonoscopy. J Cancer Educ. 10.1007/s13187-025-02608-z 40163313 10.1007/s13187-025-02608-z PMC12717180

[28] Morris NS, MacLean CD, Chew LD, Littenberg B (2006) The single item literacy screener: evaluation of a brief instrument to identify limited reading ability. BMC Fam Pract 7:21. 10.1186/1471-2296-7-21 16563164 10.1186/1471-2296-7-21 PMC1435902

[29] Spielberger CD, Gorsuch RL, Lushene R, Vagg PR, Jacobs GA (1983) Manual for the state-trait anxiety inventory. Consulting Psychologists Press, Palo Alto

[30] Prentice-Dunn S, Rogers RW (1986) Protection motivation theory and preventive health: beyond the health belief model. Health Educ Res 1:153–161. 10.1093/her/1.3.153

[31] Cardaciotto L, Herbert JD, Forman EM, Moitra E, Farrow V (2008) The assessment of present-moment awareness and acceptance: the Philadelphia mindfulness scale. Assessment 15:204–223. 10.1177/1073191107311467 18187399 10.1177/1073191107311467

[32] Lindsay EK, Creswell JD (2017) Mechanisms of mindfulness training: monitor and acceptance theory (MAT). Clin Psychol 51:48–59. 10.1016/j.cpr.2016.10.011 10.1016/j.cpr.2016.10.011 PMC5195874 27835764

[33] Lai EJ, Calderwood AH, Doros G, Fix OK, Jacobson BC (2009) The Boston bowel preparation scale: a valid and reliable instrument for colonoscopy-oriented research. Gastrointest Endosc 69:620–625. 10.1016/j.gie.2008.05.057 19136102 10.1016/j.gie.2008.05.057 PMC2763922

[34] Ahn E, Kang H (2023) Intention-to-treat versus as-treated versus per-protocol approaches to analysis. Korean J Anesthesiol 76:531–539. 10.4097/kja.23278 38031328 10.4097/kja.23278 PMC10718629

[35] Abraha I, Montedori A (2010) Modified intention to treat reporting in randomised controlled trials: systematic review. BMJ 340:c2697. 10.1136/bmj.c2697 20547685 10.1136/bmj.c2697 PMC2885592

[36] Langford AT, Resnicow K, Davis RE et al (2010) Ethnic identity predicts loss-to-follow-up in a health promotion trial. Contemp Clin Trials 31:414–418. 10.1016/j.cct.2010.06.006 20601162 10.1016/j.cct.2010.06.006 PMC3117283

[37] Butler J, Quinn SC, Fryer CS, Garza MA, Kim KH, Thomas SB (2013) Characterizing researchers by strategies used for retaining minority participants: results of a national survey. Contemp Clin Trials 36:61–67. 10.1016/j.cct.2013.05.014 23764697 10.1016/j.cct.2013.05.014 PMC3769439

